# Housing Cost Burden and Well-Being in Older Adults Moderated by Neighborhood Cohesion and Disorder

**DOI:** 10.1093/geroni/igab046.3401

**Published:** 2021-12-17

**Authors:** Ji Hyang Cheon, Min Kyoung Park, Todd Becker

**Affiliations:** 1 University of Maryland Baltimore, University of Maryland Baltimore, Maryland, United States; 2 University of Maryland Baltimore County, Baltimore, Maryland, United States; 3 University of Maryland, Baltimore, Baltimore, Maryland, United States

## Abstract

Although aging in the community promotes well-being in older adults, contextual factors (e.g., housing cost burden, neighborhood cohesion, neighborhood disorder) may impact this relationship. Identifying such risk factors represents a first step toward improving older adult well-being. NHATS data (Rounds 5–8) were used to answer two research questions (RQs). RQ1: “Is housing cost burden significantly associated with well-being?” RQ2: “Is this association further moderated by neighborhood cohesion and neighborhood disorder?” Participants were 18,311 adults ≥ 65 years old. Well-being was assessed by summing 11 commonly identified indicators. Two items were merged to assess housing cost burden (categories: “no burden,” “no money for utilities,” “no money for rent,” and “no money for utilities or rent”). Neighborhood cohesion and disorder were combined (categories: “no cohesion, no disorder,” “yes cohesion, no disorder,” “no cohesion, yes disorder,” and “yes cohesion, yes disorder”). Both RQs were assessed through a random coefficient model controlling for established covariates. RQ1 results revealed that, compared to “no burden,” “no money for utilities or rent” (B = −1.22, p = .003) and “no money for rent” (B = −1.50, p = .007) were significantly associated with well-being. RQ2 results revealed that “no cohesion, no disorder” significantly moderated the association between “no money for utilities or rent” and well-being (B = −2.44, p = .011). These results indicate that increased housing cost burden is associated with decreased well-being, especially for those reporting no neighborhood cohesion. Future research should examine neighborhood-level protective factors promoting cohesion for older adults to support well-being.

